# Prognostic Value of Transfer Learning Based Features in Resectable Pancreatic Ductal Adenocarcinoma

**DOI:** 10.3389/frai.2020.550890

**Published:** 2020-10-05

**Authors:** Yucheng Zhang, Edrise M. Lobo-Mueller, Paul Karanicolas, Steven Gallinger, Masoom A. Haider, Farzad Khalvati

**Affiliations:** ^1^Department of Medical Imaging, University of Toronto, Toronto, ON, Canada; ^2^Department of Diagnostic Imaging and Department of Oncology, Faculty of Medicine and Dentistry, Cross Cancer Institute, University of Alberta, Edmonton, AB, Canada; ^3^Department of Surgery, Sunnybrook Health Sciences Centre, Toronto, ON, Canada; ^4^Lunenfeld-Tanenbaum Research Institute, Sinai Health System, Toronto, ON, Canada; ^5^Joint Department of Medical Imaging, Sinai Health System, University Health Network, University of Toronto, Toronto, ON, Canada; ^6^Research Institute, The Hospital for Sick Children, Toronto, ON, Canada; ^7^Department of Mechanical and Industrial Engineering, University of Toronto, Toronto, ON, Canada

**Keywords:** transfer learning, radiomics, prognosis, pancreatic cancer, survival analysis

## Abstract

**Background:** Pancreatic Ductal Adenocarcinoma (PDAC) is one of the most aggressive cancers with an extremely poor prognosis. Radiomics has shown prognostic ability in multiple types of cancer including PDAC. However, the prognostic value of traditional radiomics pipelines, which are based on hand-crafted radiomic features alone is limited.

**Methods:** Convolutional neural networks (CNNs) have been shown to outperform radiomics models in computer vision tasks. However, training a CNN from scratch requires a large sample size which is not feasible in most medical imaging studies. As an alternative solution, CNN-based transfer learning models have shown the potential for achieving reasonable performance using small datasets. In this work, we developed and validated a CNN-based transfer learning model for prognostication of overall survival in PDAC patients using two independent resectable PDAC cohorts.

**Results:** The proposed transfer learning-based prognostication model for overall survival achieved the area under the receiver operating characteristic curve of 0.81 on the test cohort, which was significantly higher than that of the traditional radiomics model (0.54). To further assess the prognostic value of the models, the predicted probabilities of death generated from the two models were used as risk scores in a univariate Cox Proportional Hazard model and while the risk score from the traditional radiomics model was not associated with overall survival, the proposed transfer learning-based risk score had significant prognostic value with hazard ratio of 1.86 (95% Confidence Interval: 1.15–3.53, *p*-value: 0.04).

**Conclusions:** This result suggests that transfer learning-based models may significantly improve prognostic performance in typical small sample size medical imaging studies.

## Introduction

Pancreatic Ductal Adenocarcinoma (PDAC) is one of the most aggressive malignancies with poor prognosis (Stark and Eibl, 2015; Stark et al., 2016; Adamska et al., 2017). Evidence suggested that surgery can improve overall survival in resectable PDAC cohorts (Stark et al., 2016; Adamska et al., 2017). However, the 5-year survival rate of patients who went through surgery is still low (Fatima et al., 2010). Thus, it is important to identify high-risk and low-risk surgical candidates so that healthcare providers can make personalized treatment decisions (Khalvati et al., 2019a). In resectable patients, clinicopathologic factors such as tumor size, margin status at surgery, and histological tumor grade have been studied as biomarkers for prognosis (Ahmad et al., 2001; Ferrone et al., 2012; Khalvati et al., 2019a). However, many of these biomarkers can only be assessed after the surgery and thus, the opportunity for patient-tailored neoadjuvant therapy is lost. Recently, quantitative medical imaging biomarkers have shown promising results in prognostication of the overall survival for cancer patients, providing an alternative solution (Kumar et al., 2012; Parmar et al., 2015; Lambin et al., 2017).

**Figure 1:**
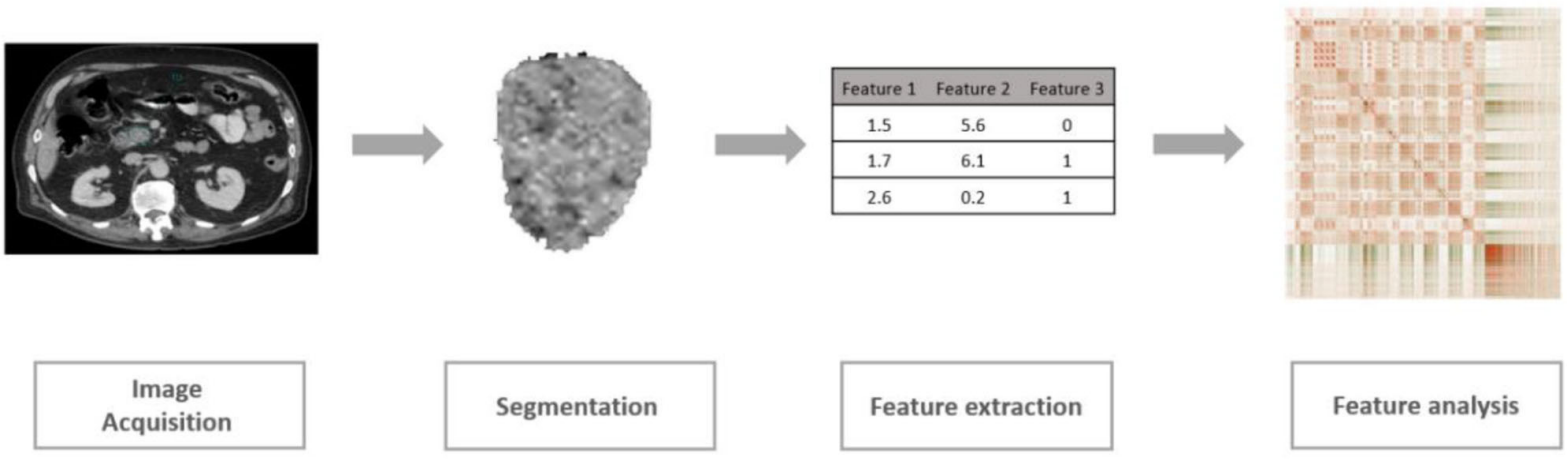
Conventional radiomics analytics pipeline.

As a rapidly developing field in medical imaging, radiomics is defined as the extraction and analysis of a large number of quantitative imaging features from medical images including CT and MRI (Kumar et al., 2012; Lambin et al., 2012; Khalvati et al., 2019b). The conventional radiomic analysis pipeline consists of four steps as shown in Figure 1. Following this pipeline, several radiomic features have been shown to be significantly associated with clinical outcomes including overall survival or recurrence in different cancer sites such as lung, head and neck, and pancreas (Aerts et al., 2014; Coroller et al., 2015; Carneiro et al., 2016; Cassinotto et al., 2017; Chakraborty et al., 2017; Eilaghi et al., 2017; Lao et al., 2017; Zhang et al., 2017; Attiyeh et al., 2018; Yun et al., 2018; Sandrasegaran et al., 2019). Using these radiomic features, patients can be categorized into low-risk or high-risk groups guiding clinicians to design personalized treatment plans (Chakraborty et al., 2018; Varghese et al., 2019). Although limited work has been done in the context of PDAC, recent studies have confirmed the potential of new quantitative imaging biomarkers for resectable PDAC prognosis (Eilaghi et al., 2017; Khalvati et al., 2019a).

Despite recent progress, radiomics analytics solutions have a major limitation in terms of performance. The performance of radiomics models relies on the amount of information that radiomics features can capture from medical images (Kumar et al., 2012). Most radiomics features represent morphology, first order, or texture information from the regions of interest (Van Griethuysen et al., 2017). The equations of these radiomic features are often manually designed. This is a sophisticated and time-consuming process, requiring prior knowledge of image processing and tumor biology. Consequently, a poor design of the feature bank may fail to extract important information from medical images, having a significant negative impact on the performance of prognostication. In contrast, the ability of deep learning for automatic feature extraction has been proven and shown to achieve promising performances in different medical imaging tasks (Shen et al., 2017; Yamashita et al., 2018; Yasaka et al., 2018).

A convolutional neural network (CNN) (Schmidhuber, 2014; LeCun et al., 2015) performs a series of convolution and pooling operations to get comprehensive quantitative information from input images (LeCun et al., 2015). Compared to hand-crafted radiomic features that are predesigned and fixed, the coefficients of CNNs are modified in the training process. Hence, the final features generated from a successfully trained CNN are tuned to be associated with the target outcomes (e.g., overall survival, recurrence). It has been shown that CNN architectures are effective in different medical imaging tasks such as segmentation for head and neck anatomy and diagnosis for the retinal disease (Dalmiş et al., 2017; De Fauw et al., 2018; Nikolov et al., 2018; Irvin et al., 2019).

However, to train a CNN from scratch, millions of parameters need to be tuned. This requires a large sample size which is not feasible to collect in most medical imaging studies (Du et al., 2018). As an alternative solution, CNN-based transfer learning is more suitable for medical imaging tasks since it can achieve a comparable performance using a limited amount of data (Pan and Yang, 2010; Chuen-Kai et al., 2015).

CNN-based transfer learning is defined as taking images from a different domain such as natural images (e.g., ImageNet) to build a pretrained model and then apply the pretrained model to target images (e.g., CT images of lung cancer) (Ravishankar et al., 2017). The idea of transfer learning is based on the assumption that the structure of a CNN is similar to the human visual cortex as both are composed of layers of neurons (Pan and Yang, 2010; Tan et al., 2018). Top layers of CNNs can extract general features from images while deeper layers are able to extract information that is more specific to the outcomes (Yosinski et al., 2014).

Transfer learning utilizes this property, training top layers using another large dataset while finetuning deeper layers using data from the target domain. For example, the ImageNet dataset contains more than 14 million images (Russakovsky et al., 2015). Hence, pretraining a model using this dataset would help the model learn how to extract general features using initial layers. Given that many image recognition tasks are similar, top (shallower) layers of the pretrained network can be transferred to another CNN model. In the next step, deeper layers of the CNN model can be trained using the target domain images (Torrey and Shavlik, 2009). Since the deeper layers are more target-specific, finetuning them using the images from the target domain may help the model quickly adapt to the target outcome, and hence, improve the overall performance.

In medical imaging, the target dataset is often so small that it is impractical to properly finetune the deeper layers. Consequently, in practice, a pretrained CNN can be used as a feature extractor (Hertel et al., 2015; Lao et al., 2017). Given that convolution layers can capture high-level and informative details from images, passing the target domain images through these layers allows extractions of features. These features can be further used to train a classifier for the target domain, enabling building a high-performance transfer learning model using a small dataset.

In this study, using two independent small sample size resectable PDAC cohorts, we evaluated the prognosis performance of a transfer learning model and compared its performance to that of a traditional radiomics model. The goal of the prognostication was to dichotomize PDAC patients who were candidates for curative-intent surgery to high-risk and low-risk groups. We found that the transfer learning model provides better prognostication performance compared to the conventional radiomics model, suggesting the potential of transfer learning in a typical small sample size medical imaging study.

## Methods

### Dataset

Two cohorts from two independent hospitals consisting of 68 (Cohort 1) and 30 (Cohort 2) patients were enrolled in this retrospective study. All patients underwent curative intent surgical resection for PDAC from 2007–2012 to 2008–2013 in Cohort 1 and Cohort 2, respectively, and they did not receive other neoadjuvant treatment. Preoperative portal venous phase contrast-enhanced CT images were used. Overall survival (including survival as duration and death as the event) was collected as the primary outcome and it was calculated as the duration from the date of preoperative CT scan until death. To exclude the confounding effect of postoperative complications, patients who died within 90 days after the surgery were excluded. Institutional review board approval was obtained for this study from both institutions (Khalvati et al., 2019a).

**Figure 2:**
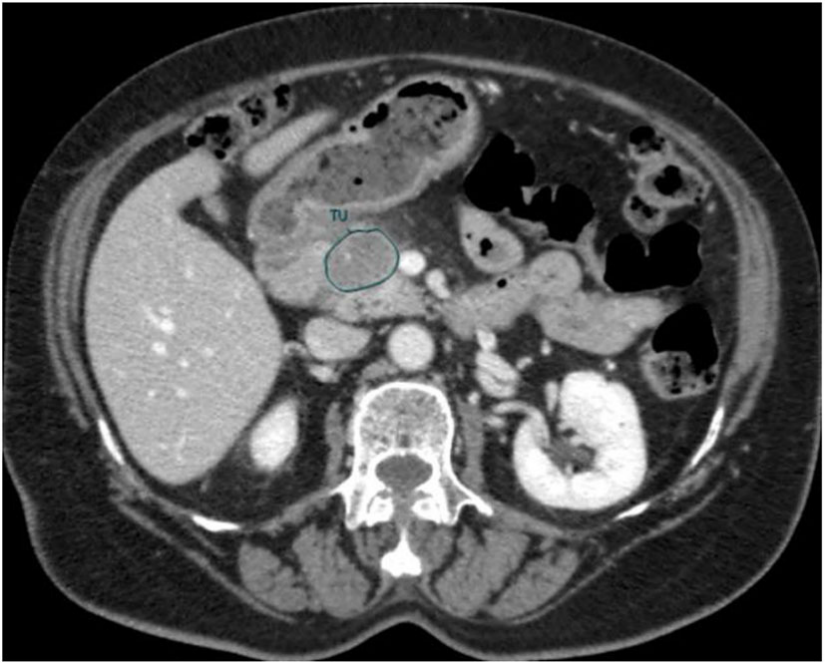
A manual contour of CT scan from a representative patient in cohort 2.

An in-house developed Region of Interest (ROI) contouring tool (ProCanVAS Zhang et al., 2016) was used by a radiologist with 18 years of experience who completed the contours blind to the outcome (overall survival). Following the protocol, the slices were contoured with the largest visible 2D cross-section of the tumor on the portal venous phase. When the boundary of the tumor was not clear, it was defined by the presence of pancreatic or common bile duct cut-off and the review of pancreatic phase images (Khalvati et al., 2019a). An example of the contour is shown in Figure 2.

### Radiomics Feature Extraction

Radiomics features were extracted using the PyRadiomics library (Van Griethuysen et al., 2017) (version 2.0.0) in Python. Voxels with Hounsfield unit under−10 and above 500 were excluded so that the presence of fat and stents will not affect the values of the features. The bin width (number of gray levels per bin) was set to 25. In total, 1,428 radiomic features were extracted from CT images within the ROI for both cohorts. Table 1 lists different classes of features used in this study (Khalvati et al., 2019a).

**Table 1:** List of radiomic feature classes and filters.

**First-order features**	**Histogram-based features**
Second-order texture features	Features extracted from Gray-Level Co-Occurrence matrix (GLCM)
Morphology features	Features based on the shape of the region of interest
Filters	No filter, exponential, gradient, logarithm, square, square-root, local binary pattern, wavelet

### Transfer Learning

**Figure 3:**
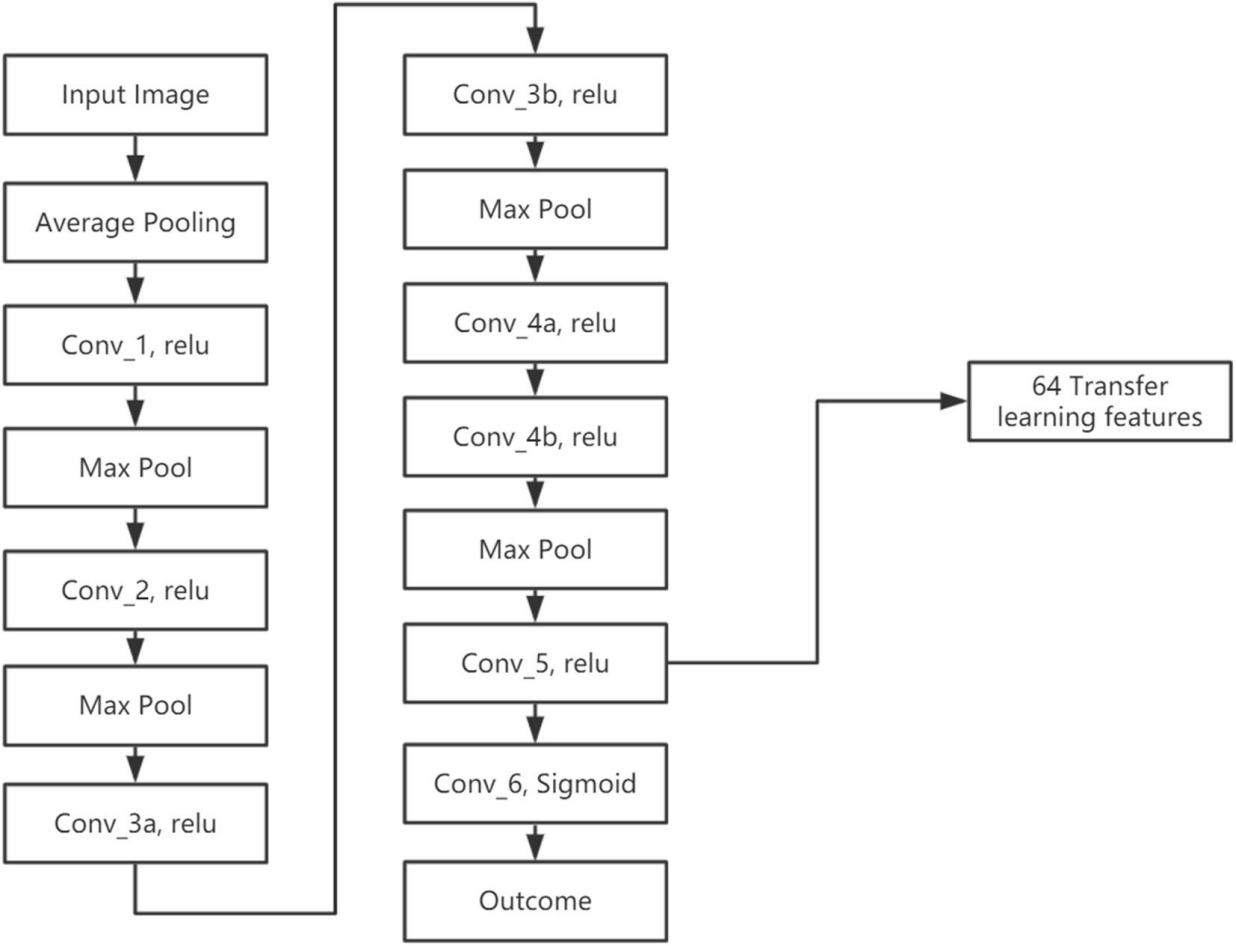
Architecture for pretrained CNN using LUNA16 data.

We developed a transfer learning model (LungTrans) pretrained by CT images from non-small-cell lung cancer (NSCLC) patients. The Lung CT dataset was published on Kaggle for Lung Nodule Analysis (LUNA16), containing CT images from 888 lung cancer patients and the outcome (malignancy or not) (Armato et al., 2011). All input ROIs were resized to 32 × 32 greyscale. An 8-layer CNN was trained from scratch using LUNA16 CT images with batch size 16 and learning rate 0.001 (Figure 3). This configuration was shown to have high performance in differentiating malignancy vs. normal tissue in the LUNA16 competition (De Wit, 2017). In addition, given small ROI sizes of data in this study (32 × 32) and the fact that images are grayscale instead of RGB color, off-the-shelf deep CNNs such as ResNet (He et al., 2015) do not provide adequate performance. Each convolutional layer except for Conv_5 has Kernel size as 3 × 3 with stride of 1 with zero padding. Conv_5 has 2 × 2 kernel size and stride of 1 without padding. All the Max Pooling layers have 2 × 2 kernel size. Previous research has shown that top layers in the CNN extract generic features from the image, while bottom layers can extract features specific to the tasks (Yosinski et al., 2014; Paul et al., 2019). Since our pretrained domain (lung CT) and target domain (PDAC CT) are rather similar, we extracted features from the bottom layer. In addition, the number of features (coefficients) in the CNN significantly decreases as the layers become deeper, due to Max pooling. If we picked a layer above the final layer, the number of extracted features would increase significantly. Considering the sample size of our training (68) and test (30) datasets, all the convolution layers were frozen and features were extracted from the end of the CNN (Conv_5). As a result, for each ROI from PDAC CT images, 64 features were extracted. This was the ideal number of intermediate features tested in LUNA16 dataset (De Wit, 2017).

### Prognostic Models

To have a proper and robust validation, training and test datasets were collected from two different institutions. In Cohort 1 (training cohort, *n* = 68), two prognostic models for overall survival were trained using features extracted from conventional radiomics feature bank (PyRadiomics) and transfer learning model (LungTrans). The prognosis models were built using the Random Forest classifier, which is a common classifier in radiomics analytic pipeline, with 500 decision trees (Chen and Ishwaran, 2012; Zhang et al., 2017). Random Forest classifier is highly data-adaptive, which have shown the potential to handle large P small N problem by choosing the best subset of features for classification (Chen and Ishwaran, 2012). The “data-adaptive” characteristic makes the random forest a good candidate for our study where transfer learning and PyRadiomics offered different numbers of features. The number of variables available for splitting at each tree node (mtry) was determined by the best performing mtry option in the training cohort. Due to the imbalanced outcome in the training data, (Cohort 1: 52 Deaths vs. 16 Survivals), a data balancing algorithm, SMOTE (Ryu et al., 2002), was applied in the training process to artificially balance the training data.

The prognostic values of these two models were evaluated in Cohort 2 (*n* = 30, 15 Deaths vs. 15 Survivals) using the area under the receiver operating characteristic (ROC) curve (AUC). DeLong test, as one of the common comparison tests, was used to test the difference between the two ROC curves (DeLong et al., 1988). To further assess the prognosis values, the predicted probabilities of death generated from the two classifiers were used as risk scores in survival analyses. These risk scores were tested in Cohort 2 using univariate Cox Proportional Hazards Model for their Hazard Ratio and Wald test *p*-value (Cox, 1972). These analyses were done in R (version 3.5.1) using “caret,” “pROC,” and “survival” packages (Kuhn, 2008; Therneau, 2020).

## Results

### Prognostic Models Performance

Using features from the PyRadiomics feature bank, the Random Forest model yielded AUC of 0.54 [95% Confidence Interval (CI): 0.32–0.76] in the test cohort (Cohort 2) (mtry: 2). In contrast, using LungTrans features, the AUC of the Random Forest model reached 0.81 (95% CI: 0.64–0.98) in the test cohort (mtry: 17). The performances of these two models for both training and test cohorts are listed in Table 2A. We performed a 5-fold cross-validation to produce AUCs for the training cohort. The AUCs for the test cohort were generated using the models trained by the training cohort.

**Table 2A:** Summary of models' performances in AUC.

	**Training cohort (*n* = 68)**	**Test cohort (*n* = 30)**
	**(5-Fold cross validation)**	
PyRadiomics model	0.57 (95% CI: 0.42–0.73)	0.54 (95% CI: 0.32–0.76)
Transfer learning model	0.72 (95% CI: 0.58–0.86)	0.81 (95% CI: 0.64–0.98)

*Tables 2B,C show Confusion Matrix for Random Forest models using PyRadiomics and LungTrans features, respectively, in the test cohort*.

**Table 2B:** Confusion Matrix of PyRadiomics model in the test cohort.

**Test cohort**	**Deceased patients**	**Survived patients**
Predicted death	12	10
Predicted survival	3	5

*Accuracy: 0.57, Sensitivity: 0.8, Specificity: 0.33, Precision: 0.55*.

**Table 2C:** Confusion matrix of transfer learning model in the test cohort.

**Test cohort**	**Deceased patients**	**Survived patients**
Predicted Death	13	4
Predicted Survival	2	11

*Accuracy: 0.80, Sensitivity: 0.87, Specificity: 0.73, Precision: 0.76*.

To investigate the prognostic value of each PyRadiomics features, variable importance indices were calculated using the Caret Package in R. The top ten features were first order entropy, first order uniformity, first order interquartile range, GLSZM gray level non-uniformity normalized, GLRLM run length non-uniformity normalized, GLCM cluster tendency, NGTDM busyness, GLSZM small area high gray level emphasis, GLSZM low gray level zone emphasis, and GLSZM large area high gray level emphasis. This confirming previous studies in this field where similar radiomic features have been reported to be prognostic of PDAC (Eilaghi et al., 2017; Chu et al., 2019; Khalvati et al., 2019a; Li et al., 2020). It is worth noting that morphologic features were not ranked as top features in the list. This may be attributed to the challenges associated with contouring the PDAC regions of interest, leading to the low robustness of morphology features.

**Figure 4:**
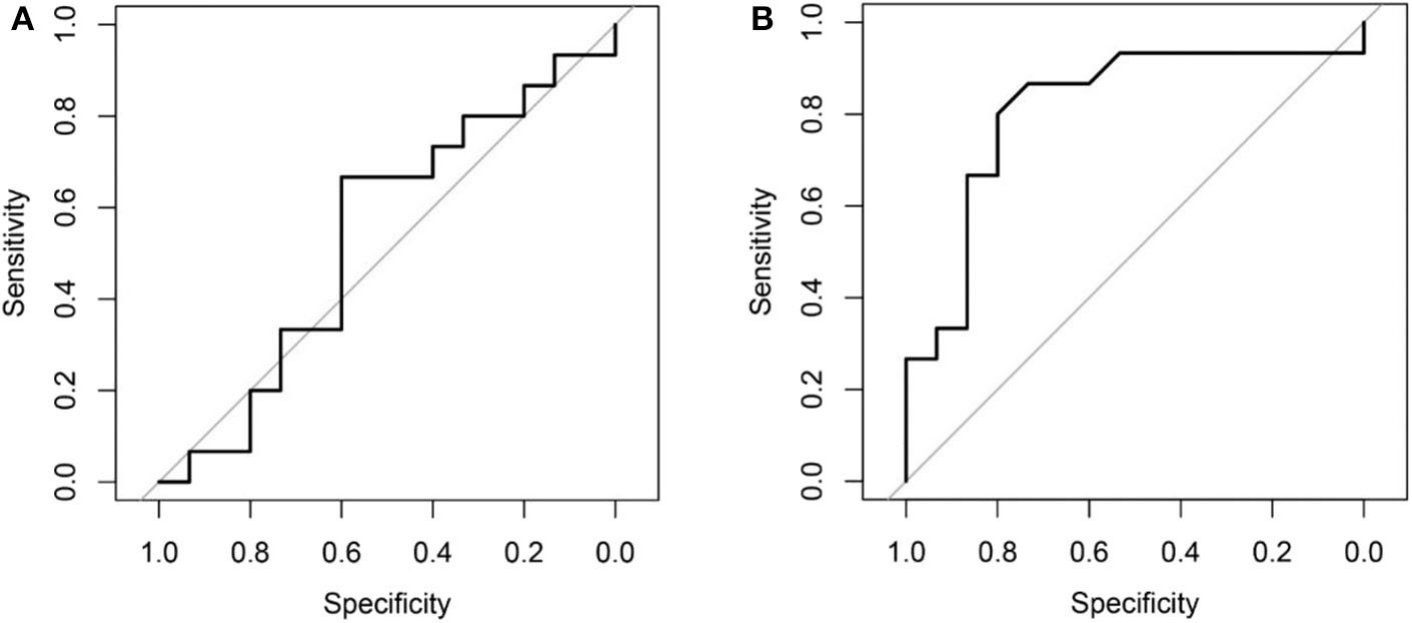
**(A)** ROC curve for the test cohort for PyRadiomics model (AUC = 0.54). **(B)** ROC curve for the test cohort for Transfer Learning (LungTrans) model (AUC = 0.81).

Comparing the ROC curves using Delong ROC test (DeLong et al., 1988), the LungTrans (Transfer Learning) prognosis model had significantly higher performance than that of PyRadiomics feature bank with a *p*-value of 0.0056 (AUC of 0.81 vs. 0.54). This result indicated that the transfer learning model based on lung CT images (LungTrans) significantly improved the prognostic performance compared to that of the traditional radiomics methods (PyRadiomics). Figure 4 shows the ROC curves for the two models for the test cohort.

### Risk Score

In univariate Cox Proportional Hazard analysis, the risk score from the PyRadiomics model was not associated with overall survival. In contrast, the risk score from the LungTrans model had significant prognostic value with a Hazard Ratio of 1.86 [95% Confidence Interval (CI): 1.15–3.53], *p*-value: 0.04 as shown in Table 3.

**Table 3:** Performance of risk score models in Cox Proportional Hazard analysis.

	**Hazard ratio and CI**	***p***
PyRadiomics based risk score	1.03 (95% CI: 0.60–1.76)	0.91
Transfer learning based risk score	1.86 (95% CI: 1.15–3.53)	0.04

**Figure 5:**
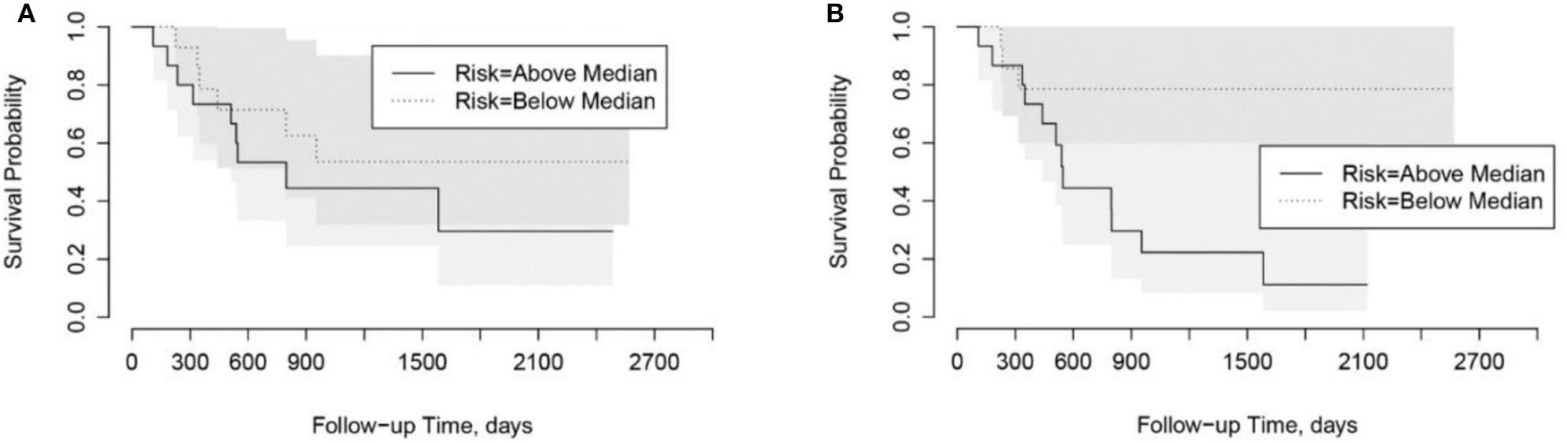
Kaplan-Meier plots for overall survival in Cohort 2. **(A)** PyRadiomics based risk score (*p* = 0.91). **(B)** Transfer Learning (LungTrans) based risk score (*p* = 0.04).

Using the risk scores, patients can be categorized into low-risk or high-risk groups based on the median values. As shown in Kaplan-Meier plots in Figure 5, the LungTrans model was able to differentiate patients with high risk from those with low risk. This result further confirms that the transfer learning feature extractor pretrained by NSCLC CT images is capable of providing prognostic information for PDAC patients.

## Discussion

In this study, we developed and compared two prognostic models for overall survival for resectable PDAC patients using the PyRadiomics and transfer learning features banks pretrained by lung CT images (LungTrans). The LungTrans model achieved significantly better prognosis performance compared to that of the traditional radiomics approach (AUC of 0.81 vs. 0.54). This result suggested that the transfer learning approach has the potential of significantly improving prognosis performance in the resectable PDAC cohort using CT images.

Previous transfer learning studies in medical imaging research often utilized ImageNet pretrained models (Chuen-Kai et al., 2015; Lao et al., 2017). In our study, we used a lung CT pretrained CNN (LungTrans) as feature extractor and showed the potential of transfer learning in a typical small sample size setting. Although CNNs are capable of achieving high performance in image recognition tasks, training these networks needs a large sample size. If a CNN with the same architecture as LungTrans was trained from scratch in the training cohort (Cohort 1), it could not provide any prognostic value in the test cohort (Cohort 2) (AUC of ~0.50). Transfer learning, unlike conventional deep learning methods which need large datasets, can achieve reasonable performance using a limited number of samples, making it suitable for most medical imaging studies. Although the training cohort in our study was small (*n* = 68), in the PDAC test cohort, our transfer learning model had positive predictive value (Precision) of 76%, demonstrating its prognostic value in finding high-risk patients. This may significantly benefit patients by providing personalized neoadjuvant or adjuvant therapy for better prognosis.

Although the proposed transfer learning model outperformed the conventional radiomics model, this was not an indication to discard radiomic features altogether. These hand-crafted features have been shown to be prognostic for survival and recurrence in different cancer sites (Kumar et al., 2012; Balagurunathan et al., 2014; Haider et al., 2017). In the PDAC radiomics field, more than forty features have been found to be significantly associated with tissue classification or overall survival for PDAC patients (e.g., sum entropy, cluster tendency, dissimilarity, uniformity, and busyness) (Cassinotto et al., 2017; Chakraborty et al., 2017; Attiyeh et al., 2018; Yun et al., 2018; Chu et al., 2019; Sandrasegaran et al., 2019; Li et al., 2020; Park et al., 2020). Furthermore, a few radiomics features have been found to be associated with tumor heterogeneity and genomics profile (Lambin et al., 2012; Itakura et al., 2015; Rizzo et al., 2016; Li et al., 2018). Hence, radiomics features can provide unique information about the lesions. Thus, studying the associations between radiomics and transfer learning features, together with feature fusion analysis, may further improve the prognostication performance in future research.

Despite achieving promising results, we should also note that the differences between NSCLC and PDAC are substantial, in terms of their biological profiles and prognoses, and thus, they may not have similar appearances in CT images. This is a limitation of the present study. A larger PDAC dataset would allow us to address these differences and test different transfer learning approaches in the context of PDAC prognosis. For example, finetuning a few layers of the CNN pretrained by NSCLS CT images using PDAC CT images would allow the network extract features that may further adapt to the PDAC images and lead to better performance.

In this study, we aimed to improve the accuracy of the survival model using the transfer learning approach. For diseases with poor prognosis, including PDAC, providing binary survival classifications offers limited information for clinicians for decision making since the survival rates are usually low. It would be more beneficial to provide time vs. risk information, e.g., identify the high-risk time intervals for a resectable PDAC patient using CT images. Future studies may choose to combine the transfer learning-based features extraction methods with the recent work on deep learning-based survival models (e.g., DeepSurv Katzman et al., 2018) to provide more practical prognosis information for personalized care.

## Conclusion

Deep transfer learning has the potential to improve the performance of prognostication for cancers with limited sample sizes such as PDAC. In this work, the proposed transfer learning model outperformed a predefined radiomics model for prognostications in resectable PDAC cohorts.

## Data Availability Statement

The datasets of Cohort 1 and Cohort 2 analyzed during the current study are available from the corresponding author on reasonable request pending the approval of the institution(s) and trial/study investigators who contributed to the dataset.

## Ethics Statement

This study was reviewed and approved by the research ethics boards of University Health Network, Sinai Health System, and Sunnybrook Health Sciences Centre. For this retrospective study the informed consent was obtained for Cohort 1 and the need for informed consent was waived for Cohort 2.

## Author Contributions

YZ, MAH, and FK contributed to the design of the concept. EML, SG, PK, MAH, and FK contributed in collecting and reviewing the data. YZ and FK contributed to the design and implementation of quantitative imaging feature extraction and machine learning modules. All authors contributed to the writing and reviewing of the paper and read and approved the final manuscript.

## Conflict of Interest

The authors declare that the research was conducted in the absence of any commercial or financial relationships that could be construed as a potential conflict of interest. The reviewer JZ declared a past co-authorship with one of the authors FK to the handling editor.

## Abbreviations

ROC: Receiver operating characteristic
AUC: Area under the ROC curve
CT: Computed tomography
CI: Confidence interval
CNN: Convolutional neural network
GLCM: Gray-Level Co-occurrence matrix
NSCLC: Non-small-cell lung cancer
PDAC: Pancreatic ductal adenocarcinoma
ROI: Region of interest
SMOTE: Synthetic minority over-sampling technique.
